# Comprehensive transcriptional landscape of porcine cardiac and skeletal muscles reveals differences of aging

**DOI:** 10.18632/oncotarget.23290

**Published:** 2017-12-15

**Authors:** Jianning Chen, Qin Zou, Daojun Lv, Yingying Wei, Muhammad Ali Raza, Yan Chen, Peilin Li, Xiaoyu Xi, Huaming Xu, Anxiang Wen, Li Zhu, Guoqing Tang, Mingzhou Li, Anan Jiang, Yihui Liu, Yuhua Fu, Yanzhi Jiang, Xuewei Li

**Affiliations:** ^1^ Department of Zoology, College of Life Science, Sichuan Agricultural University, Ya'an, Sichuan 625014, China; ^2^ Sichuan Weimu Modern Agricultural Science and Technology Co., Ltd., Chengdu, Sichuan 611130, China; ^3^ Department of Crop Cultivation and Farming System, College of Agronomy, Sichuan Agricultural University, Chengdu, Sichuan 611130, China; ^4^ Institute of Animal Genetics and Breeding, College of Animal Science and Technology, Sichuan Agricultural University, Chengdu, Sichuan 611130, China; ^5^ Sichuan Animal Husbandry Station, Chengdu, Sichuan 610041, China; ^6^ School of Computer Science and Technology, Wuhan University of Technology, Wuhan, Hubei 430070, China

**Keywords:** aging, cardiac muscle, pig, skeletal muscle, transcriptome

## Abstract

Aging significantly affects the cardiac muscle (CM) and skeletal muscles (SM). Since the aging process of CM and SM may be different, high throughput RNA sequencing was performed using CM and SM in different age conditions to evaluate the expression profiles of messenger RNA (mRNA), long non-coding RNA (lncRNA), micro RNA (miRNA), and circular (circRNA). Several mRNAs, lncRNAs, and miRNAs were highly expressed and consistently appeared in both ages in one of the two muscle tissues. Gene ontology (GO) annotation described that these genes were required for maintaining normal biological functions of CM and SM tissues. Furthermore, 26 mRNAs, 4 lncRNAs, 22 miRNAs, and 26 circRNAs were differentially expressed during cardiac muscle aging. Moreover, 81 mRNAs, 5 lncRNAs, 79 miRNAs, and 62 circRNAs were differentially expressed during aging of skeletal muscle. When comparing the expression profiles of CM and SM during aging, the senescence process in CM and SM was found to be fundamentally different. In addition, we assessed multi-group cooperative control relationships and constructed circRNA-miRNA-mRNA co-expression networks in muscular aging. In conclusion, our findings will contribute to the understanding of muscular aging and provide a foundation for future studies on the molecular mechanisms underlying muscular aging.

## INTRODUCTION

Aging affects the body's tissues in all organisms and is a major risk factor for the development of many diseases. As aging proceeds, the incidence of chronic diseases, including neurological disorders, diabetes, degenerative arthritis, and cancer increases [1]. Aging is characterized by the progressive functional decline of several organs and tissues, that eventually causes death [2]. It not only affects physical appearance of the body but also alters the expression of age-related genes [3]. In previous studies, aging-related messenger RNAs (mRNAs) were identified by analyzing the genome-wide transcriptome in humans and other mammals [4–6]. Furthermore, several conserved patterns linked to aging of the brain, such as down-regulation of genes were associated with neural functions, whereas up-regulation of genes was associated with inflammatory responses and heat shock factors [2]. Similarly, the epigenetics of aging may alter the mechanisms involved in gene expression, causing inherited DNA sequences and noncoding RNAs to contribute in defining the broad variety of aging phenotypes [7]. Several non-coding RNAs, including microRNAs (miRNAs) and long non-coding RNAs (lncRNAs), have recently gained attention because of their vital functions [8, 9]. Likewise, in some studies, the regulatory mechanisms of miRNAs [10–12] and lncRNAs [13, 14] were reported in aging mammals. Moreover, many studies documented the expression of circular RNAs (circRNAs) in both humans and mice, which were previously thought to be barely expressed [15–17]. At the cellular level, it has been confirmed that circRNAs are involved in chronic CD28-associated CD8(+)T cell aging [18]. Moreover, circRNAs were systematically expressed in skeletal muscle and were differentially expressed in advanced aged muscle, suggesting that circRNAs may affect muscle function [19]. In conclusion, circRNAs may play an important role in the regulation of aging.

Presently, one in every nine individuals worldwide is aged 60 years or older and this number is projected to increase to one in five by the year 2050. This reflects that the number of elderly people will be higher than that in the past [20], and highlights that the challenges of age-related diseases are on the rise. For example, heart failure has been reported as a serious threat to the elderly population and will continue to increase in both primary and secondary care [21]. Furthermore, slowing of processing speed is a general hallmark of human aging, which is related to the decrease in mass, strength, and contraction rate of skeletal muscle during aging [22]. The sarcopenia is primarily responsible for this decline, which becomes increasingly prevalent with age [23]. The cardiac muscle (CM) and skeletal muscle (SM) are the most important muscle tissues that perform vital body functions or support to the human body. The aging process negatively affects the ability and efficiency of CM and SM, and a decrease in mass, strength, rate of contraction and sarcopenia in SM, cardio myopathy, and cardiac fibrosis are all associated with aging [22]. Therefore, these diseases may be closely related with CM and SM. CM and SM are derived from the mesoderm layer of embryonic germ cells, which differentiate into different types of cells and have different biological functions [24], which may be result of different mechanisms of molecular regulation. Indeed, when comparing the transcriptional profiles of CM cells with that of SM cells, differences in gene expression were observed [25]. This demonstrated that aging of CM and SM may be fundamentally different, and suggested that several genes including that of mRNAs and non-coding RNAs may be involved in aging of CM and SM, whereas other mRNAs and non-coding RNAs may play a role in different age-related diseases in these muscle tissues. To our knowledge, only few studies have focused on changes of the transcriptome during CM or SM aging [26, 27], which showed that core mechanisms of cardiac aging were conserved from flies to mammals. Moreover, changes in mitochondrial metabolism and protein handling, and contractile function were conserved in hearts of Drosophila, thereby mirroring several aging processes in the mammalian hearts [26], and indicate that SM aging-related miRNAs may contribute to muscle aging with a positive regulation of transcription, and metabolic processes and kinase activity in mouse [27]. Although, these studies have expanded our knowledge on the role of age-related genes in the aging process, comprehensive changes of the transcriptome during aging of CM or SM were not studied, and no attention was paid to the differences between aging in CM and SM. Therefore, the expression profiles of mRNAs, lncRNAs, miRNAs, and circRNAs during aging of CM and SM need to be better understood, the mechanism of mutual regulation and the age-related diseases with these RNAs need to be explored, and the different regulatory mechanisms of the comprehensive transcriptional landscape in the process of aging between CM and SM needs to be revealed.

In previous studies, it was reported that pigs have a genomic structure that, except for anatomic, physiologic and biochemical similarities [28], is very similar to that of humans and mice [29], and has been found to be a valuable model for studying human diseases [30, 31]. However, only a few aging studies have been performed using pigs as an animal model. Approximately 1 years of a pig's life is equal to 5 years of human life, resulting in an average life expectancy of the pig of 15–20 years. Therefore, pigs can serve as a relevant model for studying the process of aging because of their longer lifespan compared to that of rodents and similar metabolic features, cardiovascular system, and proportional organ sizes relative to that of humans [32–34].

To better understand the transcriptional regulatory mechanism of the aging muscle, we used pigs as our animal model and compared CM and SM in older pigs at the aging stage to the CM and SM in younger pigs at maturation stage. High-throughput sequencing was used to perform an integrated multi transcriptome-wide profiling (mRNA, miRNA, lncRNA, and circRNA) analysis. This procedure enabled us to identify several differentially expressed genes or regulatory elements and unclear multi-group cooperative control networks in the aging muscle. Taken together, these data may help explain muscle age-associated changes in the transcriptional pattern during aging.

## RESULTS

### Changes in the phenotypic traits of cardiac and skeletal muscle tissues during aging

To evaluate changes in phenotypic traits of CM and SM tissues during aging, we collected CM and SM from two groups of sows (8 years old at aging stage and 180 day old young sows at maturation stage, *n* = 2 per group), that were prepared for hematoxylin-eosin staining (Supplementary Figure 1). Based on a longitudinal section of CM (10 μm), we observed that the space between muscle fibers of aged individuals was wider compared to that of younger individual (Supplementary Figure 1A). Next, we compared the area of SM fibers of young individual and aged individual, and found that the area of SM fibers of an aged individual (66.50 μm^2^) was smaller compared to that of a young individual (68.39 μm^2^) (Supplementary Figure 1B).

### Transcriptome profile of cardiac and skeletal muscles

To assess to what extent aging induces changes at the transcriptional level, we collected CM and SM from the two groups and performed transcriptome-wide profiling, including mRNA, miRNA, lncRNA, and circRNA via high-throughput sequencing. For RNA sequencing libraries, an average of ~72.14 million clean reads were obtained from each of the eight samples and 65.5%~71.8 % of these reads were uniquely aligned to the reference genome Ensemble *Susscrofa* 10.2 (Supplementary Table 1). Moreover, for small RNA sequencing libraries, approximately 9.25~17.00 million clean reads were obtained from each of the eight samples and 72.7%~90.3% of these reads were uniquely aligned to the reference genome Ensemble *Susscrofa* 10.2 (Supplementary Table 2).

**Figure 1:**
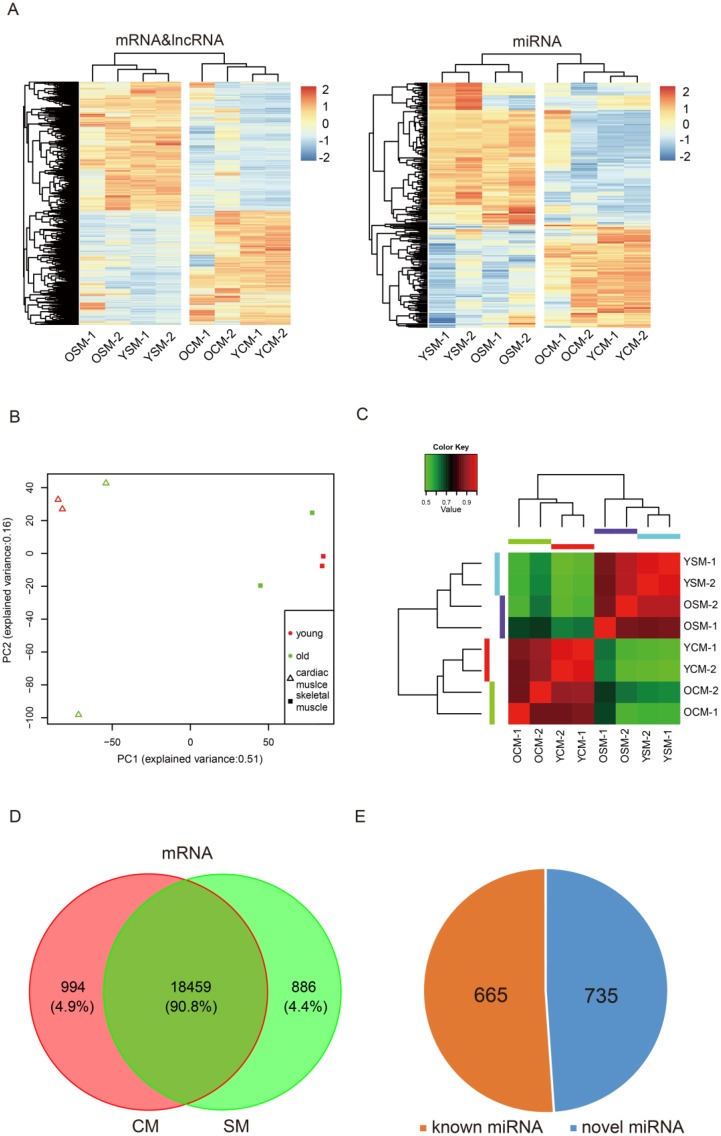
Global mRNA, lncRNA and miRNA expression pattern across samples (**A**) Heat map of expression profiles of mRNA, lncRNA and miRNA in heart and muscle. (**B**) Principal Component Analysis (PCA) plot based on normalized expression level (log2 (TPM)) of expressed mRNAs and identified lncRNAs. (**C**) Heat map of mRNA and lncRNA showing a matrix of Pearson correlation coefficient between young and old replicate samples calculated from the variance in count values where values closer to 1 are less variable. (**D**) Commonly expressed mRNA in heart and muscle. (**E**) Identified known and novel miRNA in all eight samples. Abbreviations: YCM, young cardiac muscle: OCM, old cardiac muscle; YSM, young skeletal muscle, OSM, old skeletal muscle; CM, cardiac muscle; SM, skeletal muscle.

In the samples, a total of 20339 mRNAs were detected (Supplementary Dataset 1), of which 90.8% was consistently expressed in both CM and SM. A total of 19453 and 19345 expressed mRNAs were identified in CM and SM, respectively (Figure 1A, Supplementary Dataset 1), which included 994 CM-specific and 886 SM-specific expressed mRNAs (Figure 1D, Supplementary Dataset 2). We also identified 4424 and 4327 lncRNAs in CM and SM, respectively (Supplementary Dataset 1), of which 3933 lncRNAs were consistently expressed in both muscle tissues (Supplementary Figure 1). A total of 1326 and 1303 miRNAs were identified in CM and SM, respectively (Supplementary Dataset 1), 1229 consistently expressed miRNAs were found in both tissues (Supplementary Figure 2), as well as 735 novel miRNAs (Figure 1E). In addition, we identified 7090 and 4402 circRNAs in CM and SM, respectively (Supplementary Dataset 1).

Based on the expression of the transcripts, eight samples, including old cardiac muscle (OCM) and old skeletal muscle (OSM), young cardiac muscle (YCM) and young skeletal muscle (YSM) were separated (Figure 1A), indicating tissue-specific expression patterns at the transcriptional levels. The Principal Component Analysis (PCA) showed that the mRNAs and lncRNAs transcripts from OCM or OSM had a high degree of variance between each biological replicate, whereas the YCM and YSM results were clustered (Figure 1B). PCA analyses for miRNA expression resulted in similar findings (Supplementary Figure 2A). Furthermore, we used unsupervised Euclidean matrix plots for expressed mRNA and lncRNA transcripts to determine the variance between muscle of old and young individuals (Figure 1C). The data showed that a similar tissue (CM or SM) had a closer distance than same age. Moreover, a closer distance value was found between young individuals compared to that of older individuals. A similar result was observed based on unsupervised Euclidean matrix plots of miRNAs (Supplementary Figure 2B). Thus, the results indicated that the transcriptional levels of OCM and OSM were more variable than that of their younger counterparts.

### Clustering special profiles of gene expression

**Figure 2:**
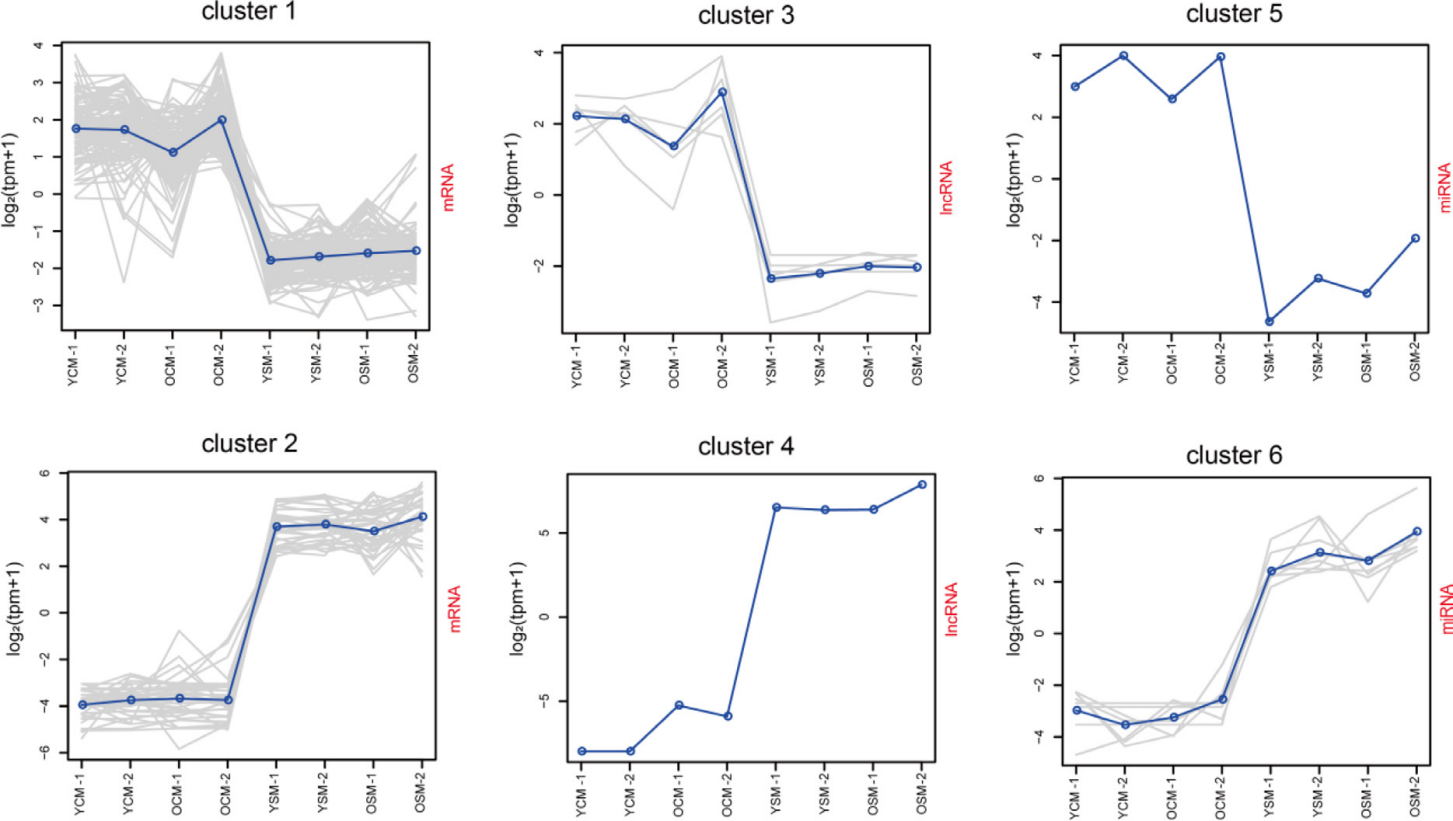
Clustering of special profiles of gene expression For each cluster, the value of the Y-axis represents gene normalized expression level (log2 (tpm+1)). The solid line represents the mean value of the cluster. The X-axis represents gene expression in the tissue. Abbreviations: YCM, young cardiac muscle; OCM, old cardiac muscle; YSM, young skeletal muscle; OSM, old skeletal muscle.

To further determine the pathways which involved in the development of the CM and SM transcriptome, we used the k-means clustering algorithm to systematically screen highly expressed RNAs, which were consistently present in both ages and identified the Gene Ontology (GO) terms with the help of the GO consortium database (described in the Methods section). After grouping 105 mRNAs in the CM-cluster 1 (Figure 2, Supplementary Dataset 3), the GO enrichment analysis showed that these genes significantly correlated to ventricular cardiac muscle tissue morphogenesis (GO: 0055010), regulation force of heart contraction (GO: 0002026), adult heart development (GO: 0007512), and regulation of heart contraction (GO: 0008016) (Supplementary Dataset 5), suggesting that expression of these genes was needed to maintain normal biological cardiac functions. In addition, 34 mRNAs were identified in the SM-cluster 2 (Figure 2, Supplementary Dataset 3) and GO analysis showed that these genes were involved in muscle contraction (GO: 0006936), sarcoplasmic reticulum (GO: 0016529), and SM fiber development (GO: 0048741) (Supplementary Dataset 5). These findings indicate that expression of these genes was vital for normal functioning of SM tissue. We further grouped 6 lncRNAs in the CM-cluster 3 (Figure 2, Supplementary Dataset 3) and only one lncRNA was present in the SM-cluster 4 (Figure 2, Supplementary Dataset 3). The co-expressed target genes of lncRNAs in CM-cluster 3 were significantly related to CM contraction (GO: 0060048) and ventricular cardiac muscle tissue morphogenesis (GO: 0055010) (Supplementary Dataset 5), whereas co-expressed target genes of lncRNA in SM-cluster 4 were involved in SM contraction (GO: 0003009) and neuromuscular synaptic transmission (GO: 0007274). In addition, we grouped 1 miRNA in CM-cluster 5 (Figure 2, Supplementary Dataset 3) and 8 miRNAs in SM-cluster 6 (Figure 2, Supplementary Dataset 3). The target genes of miRNAs in CM-cluster 5 were related to opioid receptor activity (GO: 0004985) as well as the regulation of mitochondrial membrane permeability involved in programmed necrotic cell death (GO: 1902445). The target genes of miRNAs in SM-cluster 6 referred to protein homo-oligomerization (GO: 0051260) and RNA polymerase III activity (GO: 0001056) (Supplementary Dataset 5). Thus, our results indicated that transcriptional levels in active gene regulation manifests itself as altered transcriptome profiles in a tissue-specific manner.

### Differentially expressed mRNAs during muscular aging

**Figure 3:**
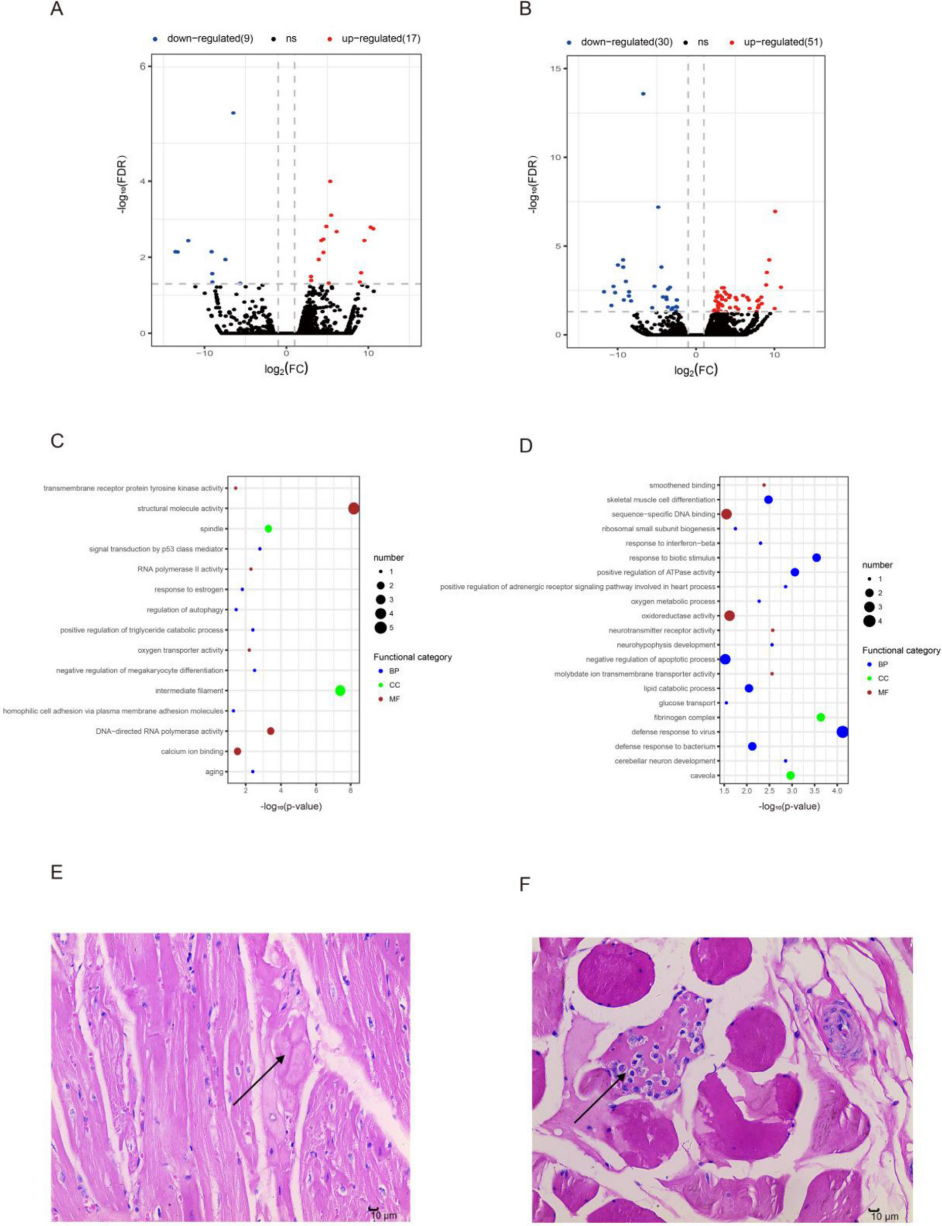
Differentially expressed mRNAs during aging (**A**) Differentially expressed mRNAs in old cardiac muscle vs. young cardiac muscle (OYCM). Red dots and blue dots represent, respectively, up-regulated and down-regulated mRNAs during aging. FDR, false discovery rate; FC, fold change. (**B**) Differentially expressed mRNAs in old skeletal muscle vs. young skeletal muscle (OYSM). (**C**) Function enrichment analysis for differentially expressed mRNAs in OYCM. Only the most enriched (*p* < 0.05) and meaningful Gene Ontology terms are presented here. BP, biological process; CC, cellular component; MF, molecular function. (**D**) Function enrichment analysis for differentially expressed mRNAs in OYSM. (**E**) Hematoxylin and eosin (HE)-stained section of old cardiac muscle (10 μm), the arrow points to hyaline degeneration. (**F**) HE-stained section of old skeletal muscle (100 μm), the arrow points to inflammatory cell infiltration.

Although several genes shared both ages in muscle development, we detected a proportion of aging-associated differentially expressed genes (DEGs) (Methods). We first screened (DEGs) in OCM vs YCM (OYCM), and identified 26 DEGs including 17 up-regulated genes and 9 down-regulated genes (Figure 3A, Supplementary Dataset 4). GO enrichment analysis showed that the up-regulated genes were related to structural proteins, triglyceride catabolic metabolic, and oxygen transport-like spindle (GO: 0005819), positive regulation of triglyceride catabolic process (GO: 0010898) and oxygen transporter activity (GO: 0005344) (Figure 3C, Supplementary Dataset 6). For example, the up-regulation of the gene *HBB* (ENSSSCG00000007978, log2FC = 5.346860054, FC = fold change) (Supplementary Dataset 4) enriched for oxygen transporter activity (Supplementary Dataset 6), which basic function was oxygen transport as one of the globin chain components of hemoglobin A [35]. Mutant *HBB* caused sickle cell anemia [36], and had a higher expression level in OCM compared to YCM, suggesting that the aging CM needs to increase oxygen transport to maintain normal levels of oxidation. Furthermore, we identified the *PNPLA2* (ENSSSCG00000012841) gene that was related to positive regulation of triglyceride catabolic processes, and was significantly up-regulated (log_2_FC = 3.03390467391754) during aging CM. Studies have shown that mutations in *PNPLA2* caused neutral lipid storage disease combined with myopathy (NLSDM) [37–41]. Down-regulated genes were related to the structural molecule, gene transcript and calcium binding capacity, such as structural molecule activity (GO: 0005198), aging (GO: 0007568), RNA polymerase II activity (GO: 0001055), and calcium ion binding (GO: 0005509) (Figure 3A, Supplementary Dataset 6). Down-regulation of the gene CDH9 (ENSSSCG00000020845, log_2_FC = -9.034974243) (Supplementary Dataset 4), which is a classic cadherin [42], was enriched for calcium ion binding. It encodes neuronal cell-adhesion [43] and had a lower expression level in OCM compared to YCM, indicating it may be related to the autonomic nervous system (controlling cardiac function), which decreases with age [44]. In addition, we observed hyaline degeneration in OCM by HE-stained muscle sections (Figure 3E). This reflected the structural changes of CM tissue during aging, which may be related to inflammaging [45].

Similarly, we obtained 81 DEGs in OSM vs YSM (OYSM), which showed 51 up-regulated genes and 30 down-regulated genes (Figure 3B, Supplementary Dataset 4). GO analysis demonstrated that these up-regulated genes were enriched for immune response, cell differentiation, metabolic process, glucose transport, and apoptotic process, such as the defense response to virus (GO: 0051607), skeletal muscle cell differentiation (GO: 0035914), lipid catabolic process (GO: 0016042), oxygen metabolic processes (GO: 0072592), glucose transport (GO: 0015758), and negative regulation of apoptotic process (GO: 0043066) (Figure 3D, Supplementary Dataset 6). We found that the genes enriched for “defense response to virus”, such as *ISG15* (log_2_FC = 6.71813789075041),*IFIT3* (log_2_FC = 5.37430318712509), *IFIT1* (log_2_FC = 5.05205229449539) (Supplementary Dataset 4), showed higher expression levels in OSM compared to YSM. Previous studies have shown that these genes were closely related to innate immunity responses [46, 47]. Moreover, over activation of ISG15 was found to uniquely associated with autoimmune disease dermatomyositis [48]. In addition, we observed inflammatory cell infiltration in HE-stained sections of OSM (Figure 3F), which may confirm that the changes in gene expression were related to “defense response to virus” tissue. Down-regulated genes were related to structural proteins, DNA binding, neuron development, response to stress and smoothened binding, such as caveolae (GO: 0005901), sequence-specific DNA binding (GO: 0043565), cerebellar neuron development (GO: 0098749), regulation of response to osmotic stress (GO: 0047484) and smoothened binding (GO: 0005119) (Figure 3D, Supplementary Dataset 6). For example, the gene *HOXA9* (ENSSSCG00000028997) (log_2_FC = -9.96363818460901) (Supplementary Dataset 4) enriched for sequence-specific DNA binding, was significantly down-regulated in SM, which confirmed that the deletion of *HOXA9* improved satellite cell function and muscle regeneration in aged mice [49].

### Differentially expressed lncRNAs during muscular aging

**Figure 4:**
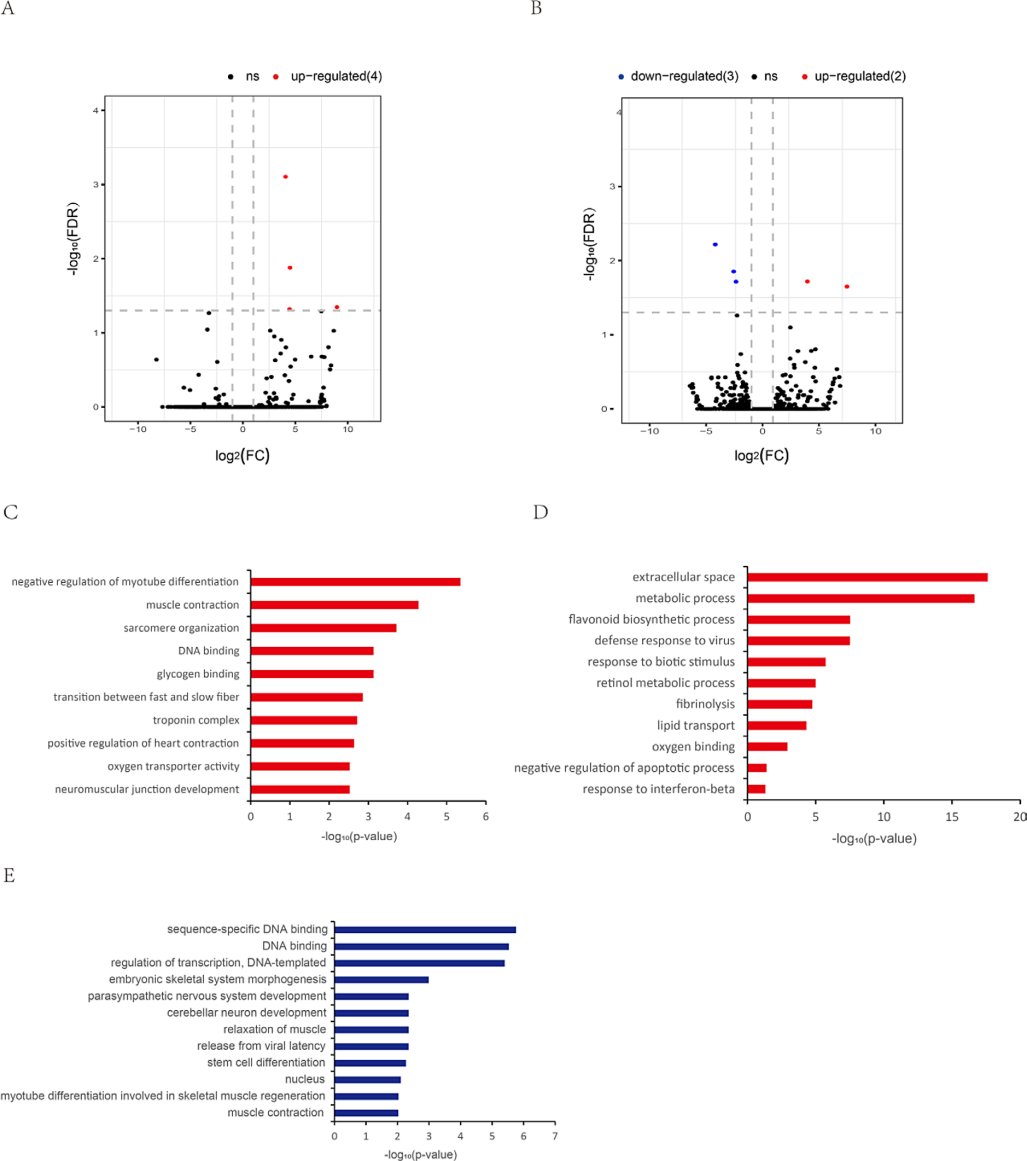
Differentially expressed lncRNAs during aging (**A**) Differentially expressed lncRNAs in old cardiac muscle vs. young cardiac muscle (OYCM). (**B**) Differentially expressed lncRNAs in old skeletal muscle vs. young skeletal muscle (OYSM). Red dots and blue dots represent, respectively, up-regulated and down-regulated mRNAs during aging. (**C**) Function enrichment analysis for targets of up-regulated lncRNAs in OYCM. Only th most enriched (*p* < 0.05) and meaningful Gene Ontology terms are presented. (**D**) Function enrichment analysis for targets of up-regulated lncRNAs in OYSM. (**E**) Function enrichment analysis for targets of down-regulated lncRNAs in OYSM.

For DELs related to aging of muscle, 9 DELs were identified in both tissues, including 4 up-regulated DELs in CM (Figure 4A, Supplementary Dataset 4), and 2 up-regulated DELs and 3 down-regulated DELs in SM (Figure 4B, Supplementary Dataset 4). GO analysis showed that these target genes of CM-DELs were significantly enriched for negative regulation of myotube differentiation (GO: 0010832) and muscle contraction (GO: 0006936) (Figure 4C, Supplementary Dataset 7). Moreover, the target genes of up-regulated SM-DELs were significantly enriched for extracellular space (GO: 0005615), metabolic process (GO: 0008152), and defense response to virus (GO: 0051607) (Supplementary Dataset 7, Figure 4D). While the target genes of down-regulated SM-DELs were closely related to DNA biding and gene transcription, such as sequence-specific DNA binding (GO: 0043565), regulation of transcription, DNA-template (GO: 0006355), some target genes were involved in cerebellar neuron development (GO: 0098749), and relaxation of muscle (GO: 0090075) (Figure 4E, Supplementary Dataset 7).

As the initial exploration of functional implications of differences in lncRNA across genomes, we investigated whether lncRNAs co-regulated the differences in target gene expression during muscle aging. Based on the results of DELs and DEGs, we investigated 7 co-expression DELs and their differentially expressed co-target genes in both tissues (Table 1). In CM, the *HBB* (DEG) was a co-target of the two up-regulated DELs (*MSTRG.51934* and *MSTRG.86430*). *HBB* was related to oxygen transport and caused sickle cell anemia by a mutation in *HBB* [35, 36]. In SM, the two up-regulated DELs (*MSTRG.212099* and *MSTRG.49389*) and their target genes were co-upregulated. *ARRDC2* is a target of *MSTRG.212099* and GO analysis showed that *ARRDC2* was involved in “cytoplasmic vesicle”. A previous study showed that *ARRDC2* was dynamically distributed throughout the plasma membrane and endocytic system, and may play a role in cargo protein trafficking within the endocytic system by mediating the discrete G protein coupled receptor (GPCR) [50]. Moreover, *IFIT3* GO enriched for “defense response to virus”, which was related to the innate immunity response, showed a maximum of Pearson correlation in four target genes of *MSTRG.49389*. In SM, the 3 down-regulated lncRNAs (*MSTRG.82735*, *MSTRG.59589* and *MSTRG.8641*) demonstrated four differentially expressed co-target genes, including *NOS1*, *OTP*, *NANOS1,* and *ENSSSCG00000026009*. Based to GO enrichment analysis, these co-target genes were related to oxidoreductase activity, neurohypophysis development, cerebellar neuron development, and receptor-mediated endocytosis. Several studies showed that *NOS1* gene polymorphism was associated with asthma [51], *OTP* played an essential role in the specification of neuronal cell lineages in the developing hypothalamus [52] and *NANOS1* was required for maintaining oocyte production [53].

**Table 1:** Gene ontology annotations of differentially expressed lncRNA and their differentially expressed targets in OHYH and OMYM

lncRNA	Targets (corrected *p*-value)	Pearson correlation	adjusted *p*-value	Gene Ontoloty annotations
OYCM				
MSTRG.51934↑	HBB (ENSSSCG00000007978)↑	0.956336221	3.87E-07	Oxygen transporter activity
MSTRG.86430 ↑	HBB (ENSSSCG00000007978)↑	0.964550884	1.07E-08	Oxygen transporter activity
OYSM				
MSTRG.212099 ↑	ARRDC2(ENSSSCG00000013893) ↑	0.975880222	9.01179E-12	Cytoplasmic vesicle
MSTRG.49389↑	IFIT3 (ENSSSCG00000010452) ↑	0.968710007	1.1554E-09	Defense response to virus
	ENSSSCG00000028736↑	0.95807849	1.94919E-07	Response to biotic stimulus
	PLAC8 (ENSSSCG00000009240)↑	0.93844449	9.74611E-05	Negative regulation of apoptotic process
	XAF1 (ENSSSCG00000017887) ↑	0.935478338	0.000200444	Response to interferon-beta
MSTRG.82735 ↓	NOS1 (ENSSSCG00000009856) ↓	0.965481669	6.71519E-09	Oxidoreductase activity
	ENSSSCG00000026009↓	0.952811701	1.40791E-06	Receptor-mediated endocytosis
	OTP (ENSSSCG00000014099) ↓	0.951857202	1.95649E-06	Neurohypophysis development
	NANOS1(ENSSSCG00000010678)↓	0.926319244	0.001461672	Cerebellar neuron development
MSTRG.59589↓	ENSSSCG00000016700↓	0.981542288	4.60321E-14	Sequence-specific DNA binding
	NANOS1 (ENSSSCG00000010678)↓	0.97617805	7.09893E-12	Cerebellar neuron development
	ENSSSCG00000026009↓	0.970619062	3.6628E-10	Receptor-mediated endocytosis
	OTP (ENSSSCG00000014099) ↓	0.949340948	4.48638E-06	Neurohypophysis development
	NOS1 (ENSSSCG00000009856) ↓	0.942962891	2.97476E-05	Oxidoreductase activity
MSTRG.8641↓	NANOS1 (ENSSSCG00000010678)↓	0.981344633	5.71122E-14	Cerebellar neuron development
	ENSSSCG00000016700↓	0.976999444	3.60652E-12	Sequence-specific DNA binding
	NOS1 (ENSSSCG00000009856)	0.96307093	2.20165E-08	Oxidoreductase activity
	ENSSSCG00000026009↓	0.950205867	3.3929E-06	Receptor-mediated endocytosis
	OTP (ENSSSCG00000014099) ↓	0.93118188	0.000530649	Neurohypophysis development

Note: Abbreviations: OYCM: old cardiac muscle vs. young cardiac muscle; OYSM: old skeletal muscle vs. young skeletal muscle. ↑: Up-regulated; ↓: Down-regulated.

### Differentially expressed miRNAs and circRNAs during muscular aging

**Figure 5:**
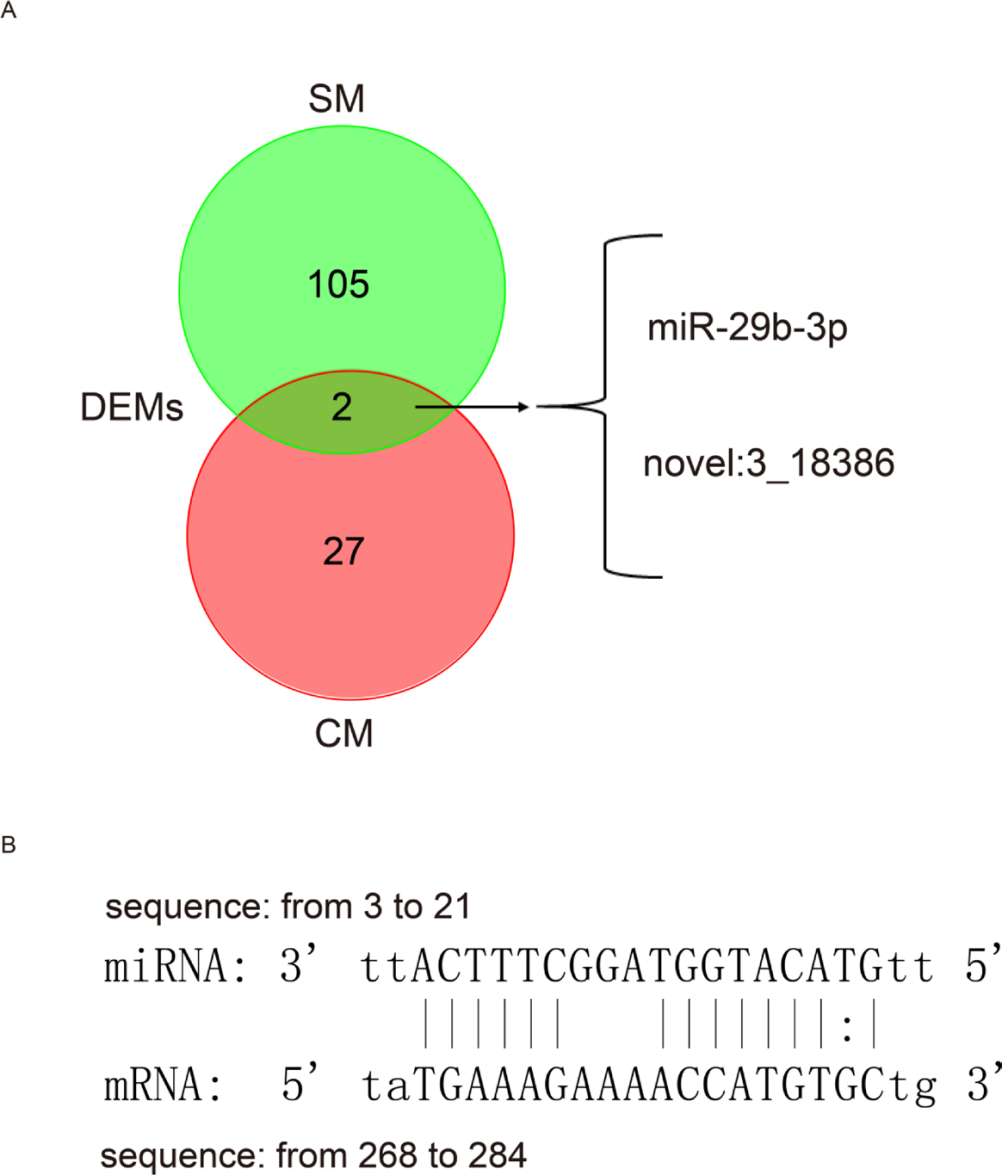
Differentially expressed miRNAs during aging (**A**) Commonly differentially expressed miRNA in both the cardiac and skeletal muscles aging process. SM, skeletal muscle; CM, cardiac muscle; DEMs, differentially expressed miRNAs. (**B**) Predicted binding position between differentially expressed *miR-493-5p* and its target *FGB* (differentially expressed) in OYSM.

A total of 22 DEMs were identified in CM of which 21 miRNAs were up-regulated and only 1 miRNAs was down-regulated (Supplementary Figure 3A, Supplementary Dataset 4). Moreover, 79 DEMs were identified in SM, of which 27 miRNAs were up-regulated and 52 miRNAs were down-regulated (Supplementary Figure 3B, Supplementary Dataset 4). Furthermore, we found that 2 miRNAs (*miR-29b-3p*, *novel: 3_18386*) were simultaneously up-regulated in both CM and SM (Figure 5A). A previous study demonstrated that *miR-29b-3p* play an important role in the formation of cardiac fibrosis, and that expression of *miR-29b-3p* significantly decreased with an increase in fibrosis marker and collagen protein. Moreover, in cardiac fibroblasts, Na/K-ATPase signaling regulates collagen synthesis through *miR-29b-3p* [54]. Here, we showed that *miR-29b-3p* was up-regulated during aging of CM and may contribute to cardiac fibrosis in the aging heart [22, 55]. Interestingly, we demonstrated that both *miR-493-5p* and its target *FGB* (Figure 5B) were significantly and differentially expressed in SM, showing that *miR-493-5p* (log_2_FC = -4.977874815) was down-regulated while *FGB* (ENSSSCG00000008997, log_2_FC = 8.954205569) was up-regulated (Supplementary Dataset 4). FGB is the beta component of fibrinogen, and when vascular injury occurs, fibrinogen is cleaved by thrombin to form fibrin, which is the most abundant component of blood clots [56].

We also identified 26 DECs in CM and of these DECs, 20 circRNAs were up-regulated while 6 circRNAs were down-regulated (Supplementary Figure 3C, Supplementary Dataset 4). Moreover, 62 DECs were identified in SM of which 40 circRNAs were up-regulated while 22 circRNAs down-regulated (Supplementary 3D, Supplementary Dataset 4). In these DECs, we found that 3 circRNAs (*circRNA020877*, *circRNA020853,* and *circRNA005658*) were simultaneously expressed in both CM and SM (Supplementary Dataset 4). The circRNA020853 was up-regulated in CM but down-regulated in SM, while *circRNA005658* was down-regulated in CM but up-regulated in SM. In addition, *circRNA020877* was shown to be down-regulated in both CM and SM.

### Construction of the circRNA-miRNA-mRNA co-expression network

**Figure 6:**
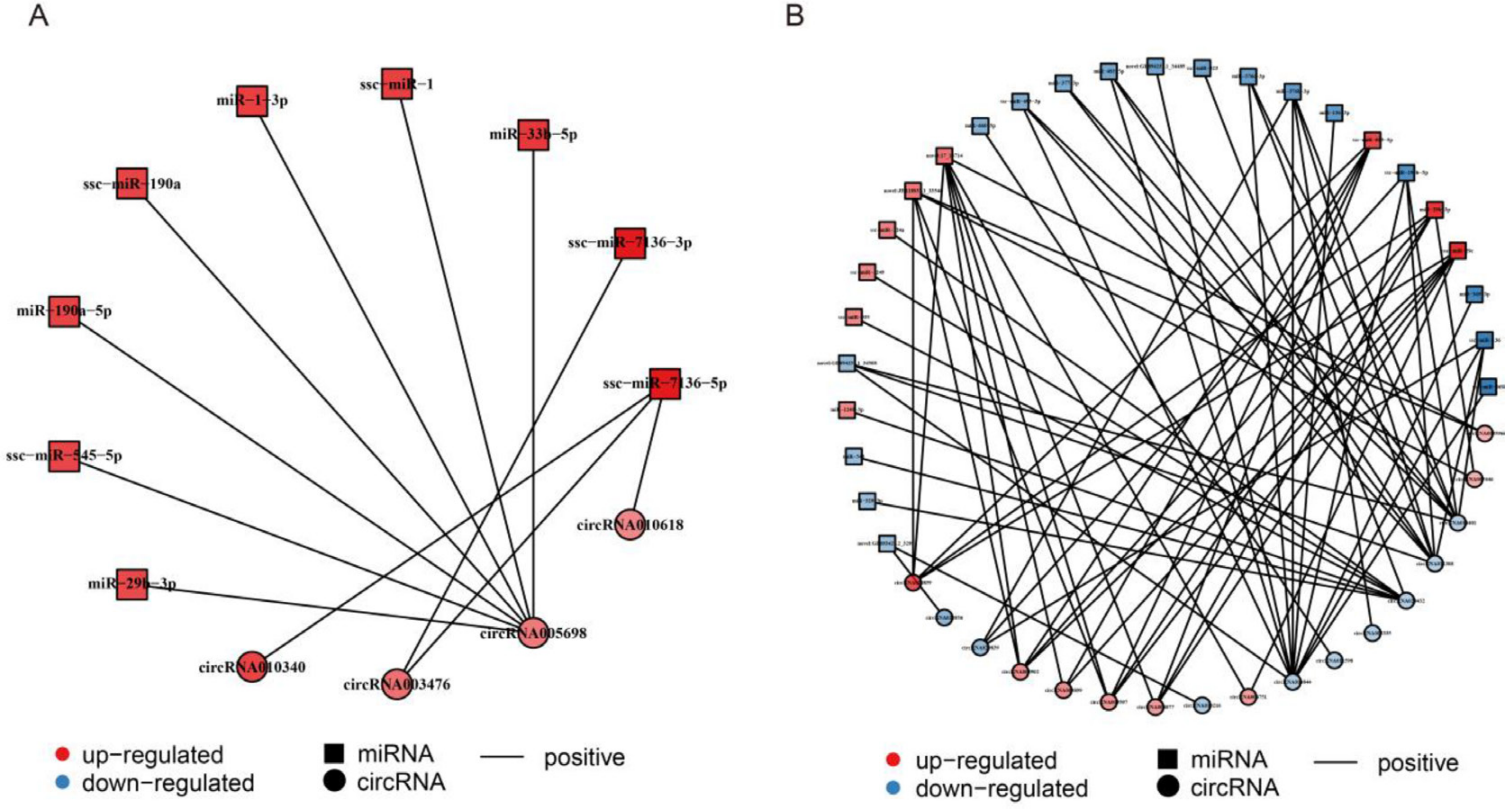
Construction of the circRNA-miRNA co-expression network (**A**) Construction of the circRNA-miRNA co-expression network in cardiac muscle (CM). (**B**) Construction of the circRNA-miRNA co-expression network in skeletal muscle (SM). Differentially expressed miRNA and circRNA were used to construct the circRNA-miRNA co-expression network in CM and SM. Square nodes represent differentially expressed miRNAs and circle nodes represent differentially expressed circRNAs. Red color and blue color represent up and down regulation, respectively. The black solid line represents the positive correlation.

It has been shown that circRNAs may act as competing endogenous RNAs (ceRNAs), which regulate the function of miRNAs [57, 58], suggesting that circRNAs and their target miRNAs may be co-expressed in the aging muscle. The co-expression network between circRNAs and their target miRNAs were predicted by the Pearson correlation coefficient and based on DECs and DEMs data [59]. For CM, a network map was constructed, which contained 9 DEMs, 4 DECs, and 11 relationships (Figure 6A, Supplementary Dataset 8). Within the network, all miRNAs were co-related with simultaneous up-regulation of circRNAs; *circRNA005698,* with 7 miRNAs, showing most relations. Similarly, a complex network map was constructed for SM, which contained 26 DEMs, 17 DECs, and 69 relationships (Figure 6B, Supplementary Dataset 8). In the network, several up- or down-regulated miRNAs were co-related with up- or down-regulated circRNAs, and *circRNA014844,* with 11 miRNAs, showing the most relations.

**Figure 7:**
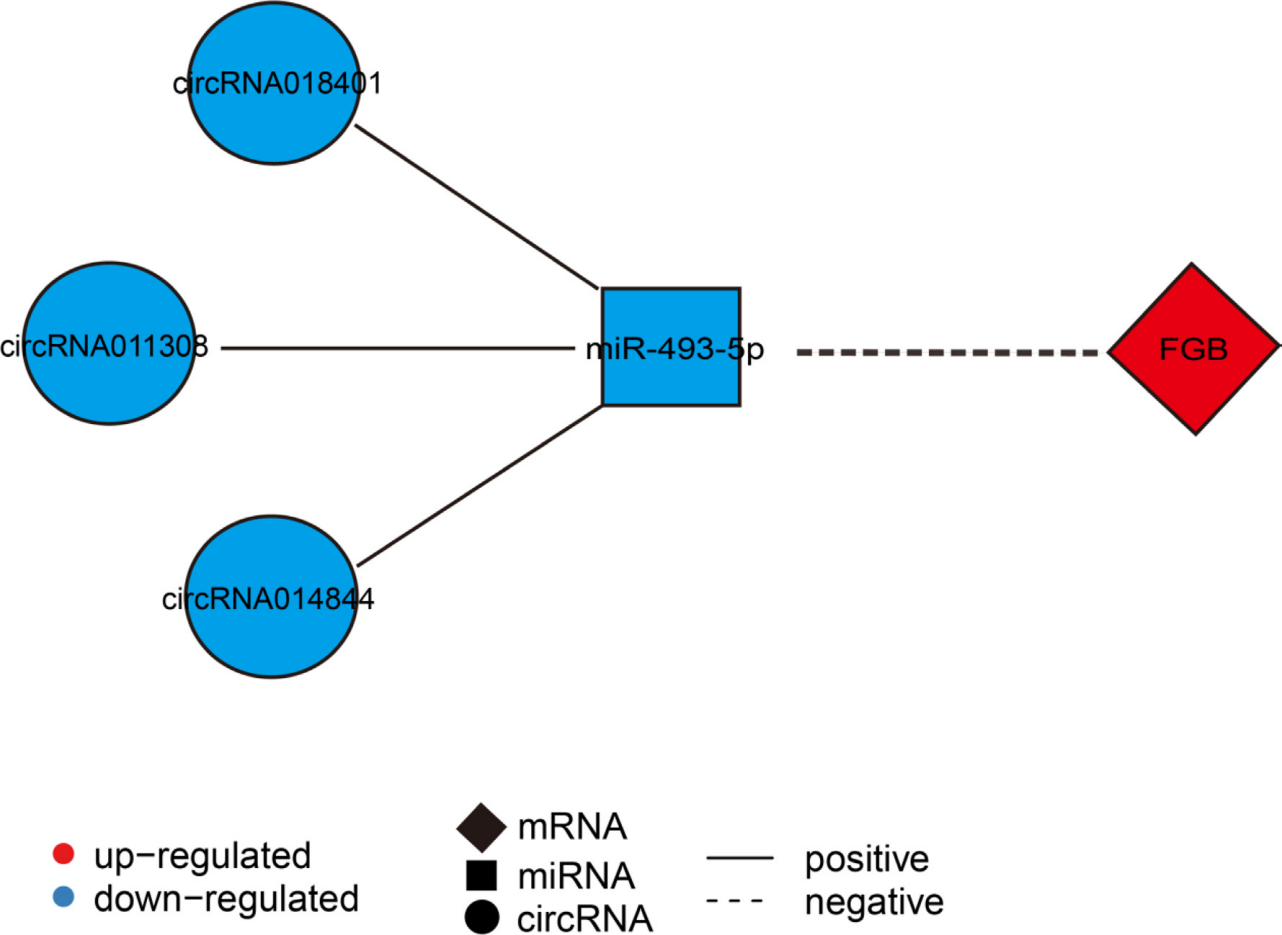
Construction of the mRNA-miRNA-circRNA interaction network An mRNA-miRNA-circRNA interaction network was constructed between FGB, has-miR-493-3p, and three circRNAs (*circRNA014844*, *circRNA011308,* and *circRNA018401*). Diamond nodes represent differentially expressed mRNA, square nodes represent differentially expressed miRNA, and circle nodes represent differentially expressed circRNAs. Red color and blue color represent up and down regulation, respectively. The black solid line represents a positive correlation, whereas the black dotted line represents a negative correlation.

These findings prompted us to further analyze the circRNA-miRNA-mRNA co-expression network. In the network, we found that *FGB* could be targeted by *miR-493-5p*, suggesting that it may be a crucial factor mediated by circRNAs-*miR-493-5p* axes. According to the DECs, DEMs, and DEGs data, we created the circRNA (*circRNA014844*, *circRNA011308,* and *circRNA018401*)-*miR-493-5p*-*FGB* co-expression pathway in SM (Figure 7). In the network, the down-regulated *miR-493-5p* was positively co-related with three down-regulated circRNAs (*circRNA014844*, *circRNA011308,* and *circRNA018401*), while it was negatively co-related with the up-regulation of its target gene *FGB*.

### The expression of genes validated by q-PCR

Here, we performed q-PCR to validate the high throughput sequencing results. We selected 9 differentially expressed genes to performed q-PCR, including 3 mRNAs, 2 lncRNAs, 2 miRNAs, and 2 circRNAs (Supplementary Figure 4). Expression of several selected genes was significantly different during aging, which may be closely related to biological functions or function in a network, such as *PNPLA2, HOXA9*, *FGB* (Supplementary Figure 4A), lncRNA *MSTRG.8641* (Supplementary Figure 4B), miRNA *miR-493-5p* (Supplementary Figure 4C), and circRNA *circRNA014844* (Supplementary Figure 4D). Similarly, several genes were randomly selected from the differentially expressed genes, including *MSTRG.143042* (Supplementary Figure 4B), *miR-29b-3p* (Supplementary Figure 4C), and *circRNA010340* (Supplementary Figure 4D). The data indicated that the expression patterns were highly consistent between the two methods (Supplementary Figure 4), for example, according to the high throughput sequencing, *miR-29b-3p* was up-regulated in both aging CM and SM, and the expression patterns were highly consistent with that of the q-PCR results. The q-PCR results confirmed the high reproducibility and reliability of the gene expression profiling in our study.

## DISCUSSION

To our knowledge, this is the first time that the profiles of transcriptome expression were comprehensively analyzed and the differences compared between CM and SM during aging. In our study, we found significant differences in gene expression for CM and SM. Several genes were highly expressed in one tissue and hardly expressed in the other (Figure 1A), indicating the different functions at a general transcriptome level in these muscle types. In addition, we identified genes for tissue-specific expression, and found that 4.9% of 20339 genes (*n* = 994) were expressed in CM and 4.4% (*n* = 886) in SM (Figure 1D). Furthermore, we analyzed these genes and identified that several highly expressed genes did not show an obvious change during aging. Indeed, GO annotation showed these genes were required for maintaining normal biological function of CM and SM. This provides new information through which we can view the differences in functions of CM and SM at the mRNA, lncRNA, and miRNA levels.

In our study, we demonstrated an increased individual variation by analyzing mRNAs and lncRNAs (Figure 1B) in CM and SM during aging. Similar findings were found in aging mouse liver by analyzing mRNAs [60]. Additionally, we examined miRNAs to verify this variation (Supplementary Figure 2A) and similar results were obtained. Moreover, this was the first time to report variations of lncRNA and small RNA, indicating that increased individual variation during aging was a common feature that not only reflected multiple organizations but also reflected multi-layer of the transcriptome.

We identified several age-related genes for CM and SM during aging and found more up-regulated genes compared to down-regulated genes. These results were in line with similar findings reported in previous studies [26, 60–62]. Perhaps, when the body ages the ability of several metabolic functions decreased and could be compensated by increasing associated genes expression. During aging, we found a higher number of differentially expressed genes in SM compared to CM, which may be related to fact that SM performs more complex biological functions than CM.

We found that differentially expressed genes were closely related to the biological function of tissue and certain diseases. Here, we found that the *PNPLA2* (ENSSSCG00000012841) gene was related to positive regulation of triglyceride catabolic process and was significantly up-regulated (log_2_FC = 3.03390467391754) during CM aging. Previous studies reported that mutations in PNPLA2 could cause neutral lipid storage disease with myopathy (NLSDM) [37–41]. This implied that *PNPLA2* expression increased during aging of the heart, and improved triglyceride metabolism which may be important for preventing NLSDM in the process of biological aging. We found that *HOXA9*, which is related to sequence-specific DNA binding (Figure 3D, Supplementary Dataset 6), was significantly down-regulation ((log_2_FC = -9.96363818460901)) in SM during aging, and was negligibly expressed in older muscle tissue. In a previous study, it was demonstrated that the inhibition of aberrant chromatin activation or deletion of *HOXA9* improved the satellite cell, which can differentiate and merge to form new mature multi-nucleated muscle cells [63], function and muscle regeneration in aged mice [49]. Likewise, *HOXA9* significantly declined in SM, which may indicate that the SM needs to increase the rate of muscle regeneration to repair damaged muscle fibers during aging. However, the capability of muscle reconstruction decreases at a later age [64].

In our study, we found that *miR-493-5p* and the target of *miR-493-5p* decreased and increased, respectively during SM aging. Further, according to the co-expression network of circRNA-miRNA, we found that *circRNA014844*, *circRNA011308,* and *circRNA018401* were closely related to *miR-493-5p* expression. Therefore, we constructed an mRNA-miRNA-circRNA interaction network, which showed that *FGB* is related to procoagulation [56]. Therefore, we speculated that as the muscle tissue ages, procoagulation in the muscle may become weak and more fibrinogen needs to be produced to accelerate the generation of blood clots. *FGB*, *miR-493-5p,* and circRNAs may play a key role in regulating procoagulation during aging. Similarly, studies have shown that mutations in the *FGB* gene could lead to afibrinogenemia [65–67], and FGB tyrosine nitration is a prothrombotic risk factor [68]. A higher thrombotic risk in elderly was partially due to a higher procoagulation state [69]. Thus, the increased *FGB* during aging found in our study may be contributed to this risk.

## MATERIALS AND METHODS

### Animals

Four healthy female Yana pigs (an indigenous Chinese pig) were used in this study. These pigs included two 180 day-old young sows and two 8 year-old sows. There were no direct and collateral blood relationships within the last 3 generations among the 4 pigs. The piglets were weaned at 28 ± 1 day of age. A starter diet was administered from day 30 to day 60 after weaning, and included 3.40 Mcal kg^−1^ metabolizable energy with 20.0% crude protein (11.5 g/kg lysine). From day 61 to day 120, the pigs were fed a diet, containing 14.0 MJ/kg of metabolizable energy consisting of 18.0% crude protein (9.0 g/kg lysine). From day 121, pigs received a diet containing 13.5 MJ/kg of metabolizable energy and 16.0% crude protein (8.0 g/kg lysine). Animals were allowed access to food and water *ad libitum* and were maintained under the same conditions. The night before slaughtering, pigs were fastened and given two hours for rest after transportation. Then, pigs were stunned electrically at 90 V and 50 Hz for 10 s, and exsanguinated as essential to ameliorate pain.

### Samples preparation

All samples used in this study were collected according to the guidelines for care and use of experimental animals established by the Ministry of Science and Technology of China. From each pig, the following tissues were collected: CM, *longissimus dorsi muscle* (LDM), SM. Tissues were rapidly separated from each carcass, immediately frozen in liquid nitrogen, and stored at -80°C until RNA extraction. Total RNA was extracted using TRIzol Reagent (Invitrogen, CA, USA), and treated with DNase and purified using an RNeasy Mini Kit (Qiagen, Valenica, CA, U.S.A.). The concentration and quality of RNA was assessed on the Agilent Bioanalyzer 2100 system.

### RNA sequencing and data analysis

A total of 5 μg RNA per sample was used as input material for the RNA sample preparations. Sequencing libraries were generated using the rRNA-depleted RNA by NEBNext^®^ Ultra™ Directional RNA Library Prep Kit for Illumina^®^ ((New England Biolabs Ipswich, Massachusetts, USA) following the manufacturer's guidelines and library quality was assessed using an Agilent Bioanalyzer 2100 system. After cluster generation, libraries were sequenced on an Illumina HiSeq 4000, and 150 bp paired-end reads were generated. Clean reads were obtained after removing reads containing the adapter, reads containing ploy-N and low quality reads from raw data. Clean reads were aligned to the Ensemble (Susscrofa 10.2) using TopHat2 (v2.0.14) [70] with default parameters.

StringTie software [71] was used to assemble the mapped reads of each sample, which at least existed in one of both replicates. Transcripts included BLASTed (evalue = 1e-10) to Ensembl, and mapped transcripts were directly described as known lncRNA or mRNA. Here, we used Salmon (v0.6.0) [72] to calculate transcripts per million (TPMs) of both lncRNAs and coding genes in each sample. Then, Coding Potential Calculator (CPC) (0.9) [73] and Pfam Scan (v1.5) [74] were performed to analyze the coding potential of transcripts. Transcripts predicted to have coding potential by either/all of the tow tools were filtered out, and those without coding potential served as our candidate set of novel lncRNAs.

### Small RNA sequencing and data analysis

A total of 5 μg total RNA per sample was used as input material for the small RNA library. Sequencing libraries were generated using NEBNext^®^ Multiplex Small RNA Library Prep Set for Illumina^®^ ((New England Biolabs Ipswich, Massachusetts, USA) following the manufacturer's guidelines, and index codes were added to attribute sequences to each sample. Library quality was assessed using an Agilent Bioanalyzer 2100 system. The clustering of the index-coded samples was performed on a cBot Cluster Generation System using TruSeq SR Cluster Kit v3-cBot-HS (Illumina, Inc, California) according to the manufacturer's guidelines. After cluster generation, the library preparations were sequenced on an Illumina MiSeq platform, and 50 bp single-end reads were generated.

The miRBase21 was used as reference, and software mirdeep2 [75] was used to obtain the potential miRNA, and to predict novel miRNA. In detail, we used miRanda (v3.3a) software [76] with default parameters and cutoffs (Score S ≥ 140 and Energy E ≤– 20.0) to predict miRNA target. Subsequently, miRNA expression levels were estimated by TPMs by the following criteria: normalization formula: normalized expression = mapped read count/total reads*1000000.

### Clustering profiles of gene expression

In this study, the k-means clustering algorithm was applied to analyze the expressed genes. For each cluster [77], gene normalized expression level was presented in log_2_ (tpm+1).

### Differential expression analysis

Differentially expressed mRNAs, lncRNAs, miRNAs and circRNAs were discovered using the edgeR package [78] running in the R programming environment. The edgeR user guide was followed as detailed in the “classic analysis” section. A differential expression *q*-value < 0.05 and fold change > 2 assigned as differentially expressed in two different comparisons (OCM vs. YCM, OSM vs. YSM). For DECs, we used a standard *p*-value < 0.05 and fold change > 2, fold change was log2 transformed ratio of average expression between two groups, calculated by edgeR.

### Gene ontology enrichment analysis

GO (Gene Ontology) enrichment analysis was implemented by the GOseq R package, GO terms with corrected *p*-value < 0.05 were considered significantly enriched by differentially expressed genes.

### CircRNA-miRNA co-expression network

The circRNA-miRNA co-expression network was constructed based on the correlation analysis between the differentially expressed circRNA and miRNAs. The expressions of differentially expressed circRNAs and miRNAs were analyzed by Pearson's correlation coefficient. The absolute coefficient value of 0.8 between a circRNA and a miRNA was considered relevant for network construction. A *p*-value of < 0.05 was considered statistically significant.

### CircRNA-miRNA-mRNA co-expression network

The mRNA-miRNA interactions were predicted by miRanda (v3.3a), Minimum Free Energy (MFE) threshold values for miRanda was set to -20 kcal/mol. We first analyzed the results of mRNA-miRNA and circRNA-miRNA co-expression network, then constructed the circRNA-miRNA-mRNA co-expression network.

### Quantitative PCR validation

cDNA was synthesized using the oligo (dT) and random 6-mer primers provided in the PrimeScript RT Master Mix kit (TaKaRa, Shiga, Japan). q-PCR was performed using the SYBR Premix Ex Taq kit (TaKaRa, Shiga, Japan) on a CFX96 Real-Time PCR detection system (Bio-Rad Laboratories, Inc., Hercules, CA, U.S.A.). The q-PCR validation was carried out using three biological replicates. The primer pairs used are presented in Supplementary Table 3. Three endogenous control genes (porcine *GAPDH*, *ACTB,* and *U6* snRNA) were used in this assay. The ΔΔCt method was used to determine the expression level of objective mRNAs, miRNAs, lncRNAs, and circ RNAs.

### Ethics approval and consent to participate

All experiments involving animals were conducted according to the Regulations for the Administration of Affairs Concerning Experimental Animals (Ministry of Science and Technology, China, revised in June 2004), and approved by the Institutional Animal Care and Use Committee in College of Animal Science and Technology, Sichuan Agricultural University (Sichuan, China) under permit No. SKY-B20160201.

## CONCLUSIONS

In summary, in the present study the known expression profiles of transcriptome for CM and SM are explored during aging. We grouped several tissue-specific high expression genes at both ages, and analysis results indicated that transcriptional levels in active gene regulation manifests itself and altered transcriptome profiles in a tissue-specific manner. Furthermore, we found that several differentially expressed genes were closely related to some serious health conditions and that may several genes may play a crucial role in regulating the functions during aging. We also compared the expression profiles of CM and SM during aging and found significant differences between the two muscle types, indicating the senescence process of CM and SM is fundamentally different. In addition, we constructed the circRNA-miRNA-mRNA co-expression networks in muscular aging cycle. Our findings contribute to the understanding of muscular aging and provide a foundation for future studies of the molecular mechanisms underlying muscular aging.

## SUPPLEMENTARY MATERIALS FIGURES AND TABLES



















## Abbreviations

HBB: hemoglobin subunit beta
PNPLA2: patatin like phospholipase domain containing 2
CDH9: cadherin 9
ISG15: ubiquitin-like modifier
IFIT3: interferon induced protein with tetratricopeptide repeats 3
IFIT1: interferon induced protein with tetratricopeptide repeats 1
HOXA9: homeobox A9
ARRDC2: arrestin domain containing 2
NOS1: nitric oxide synthase 1
OTP: orthopedia homeobox
NANOS1: nanos C2HC-type zinc finger 1
FGB: fibrinogen beta chain
GAPDH: glyceraldehyde-3-phosphate dehydrogenase
ACTB: actin beta
