# Supplementary material for: Comprehensive transcriptional landscape of porcine cardiac and skeletal muscles reveals differences of aging
Source: Oncotarget. 2017 Dec 15;9(2):1524–41. doi: 10.18632/oncotarget.23290 (PMC5788579; doi:10.18632/oncotarget.23290)
Supplement: Supplementary file 1 [file oncotarget-09-1524-s001.pdf]

Comprehensive transcriptional landscape of porcine cardiac and skeletal muscles reveals differences of aging

SUPPLEMENTARY MATERIALS

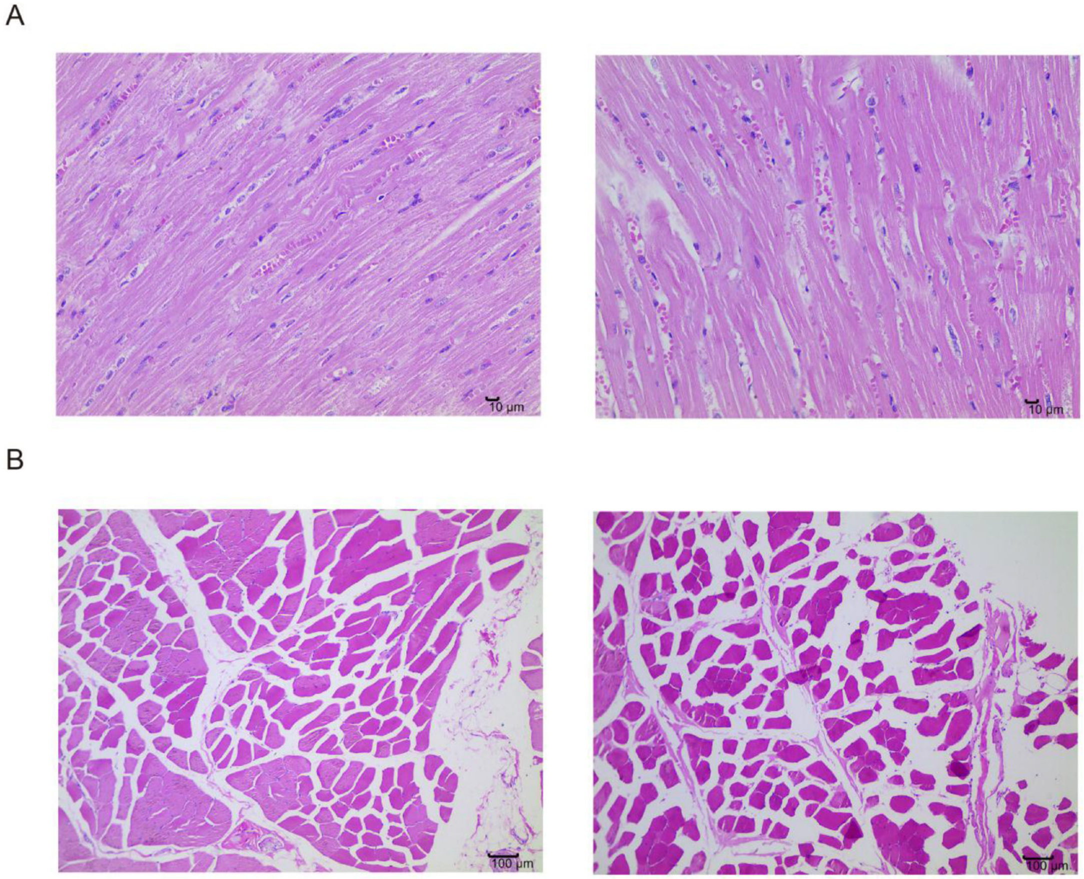

Supplementary Figure 1: Hematoxylin and eosin staining of two muscle tissues at two different age stages. (**A**) Left figure: longitudinal section of OCM (10 μm). Right figure: longitudinal section of OCM (**B**) Left figure: cross section of YSM (100 μm). Right figure: cross section of YSM (100 μm). "YCM" is short for "young cardiac muscle", "OCM" is short for "old cardiac muscle"; "YSM" is short for "young skeletal muscle", "OSM" is short for "old skeletal muscle".

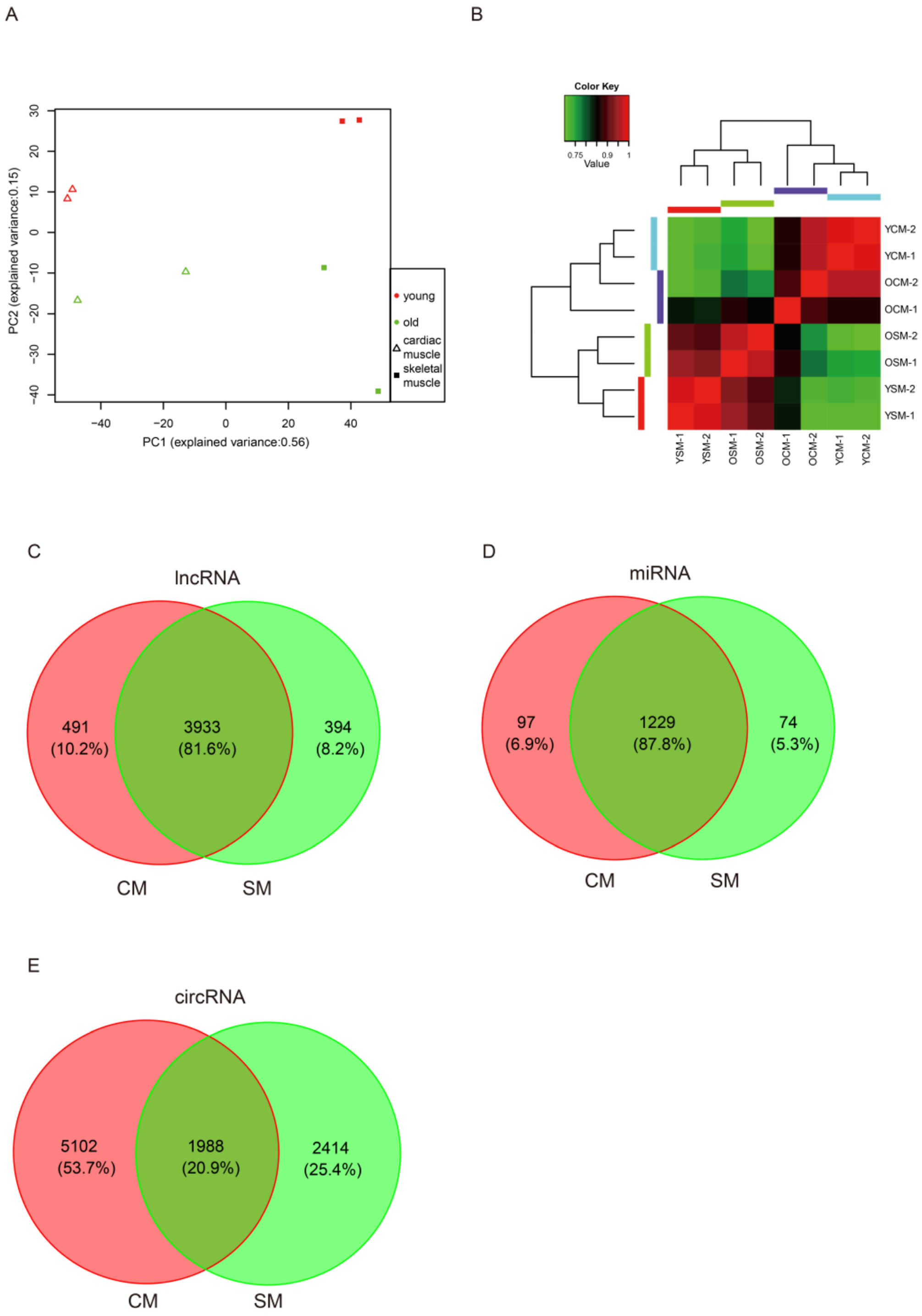

Supplementary Figure 2: Global lncRNA, miRNA, and circRNA expression pattern across samples. (**A**) Principal Component Analysis (PCA) plot based on normalized expression level (log2 (TPM)) of identified miRNAs. (**B**) Heatmap of miRNA showing matrix of Pearson correlation coefficient between young and old replicate samples calculated from the variance in count values where values closer to 1 are less variable. Common expressed lncRNA (**C**), miRNA (**D**) and circRNA (**E**) in CM and SM. "YCM" is short for "young cardiac muscle", "OCM" is short for "old cardiac muscle"; "YSM" is short for "young skeletal muscle", "OSM" is short for "old skeletal muscle"; "CM" is short for "cardiac muscle"; SM" is short for "skeletal muscle".

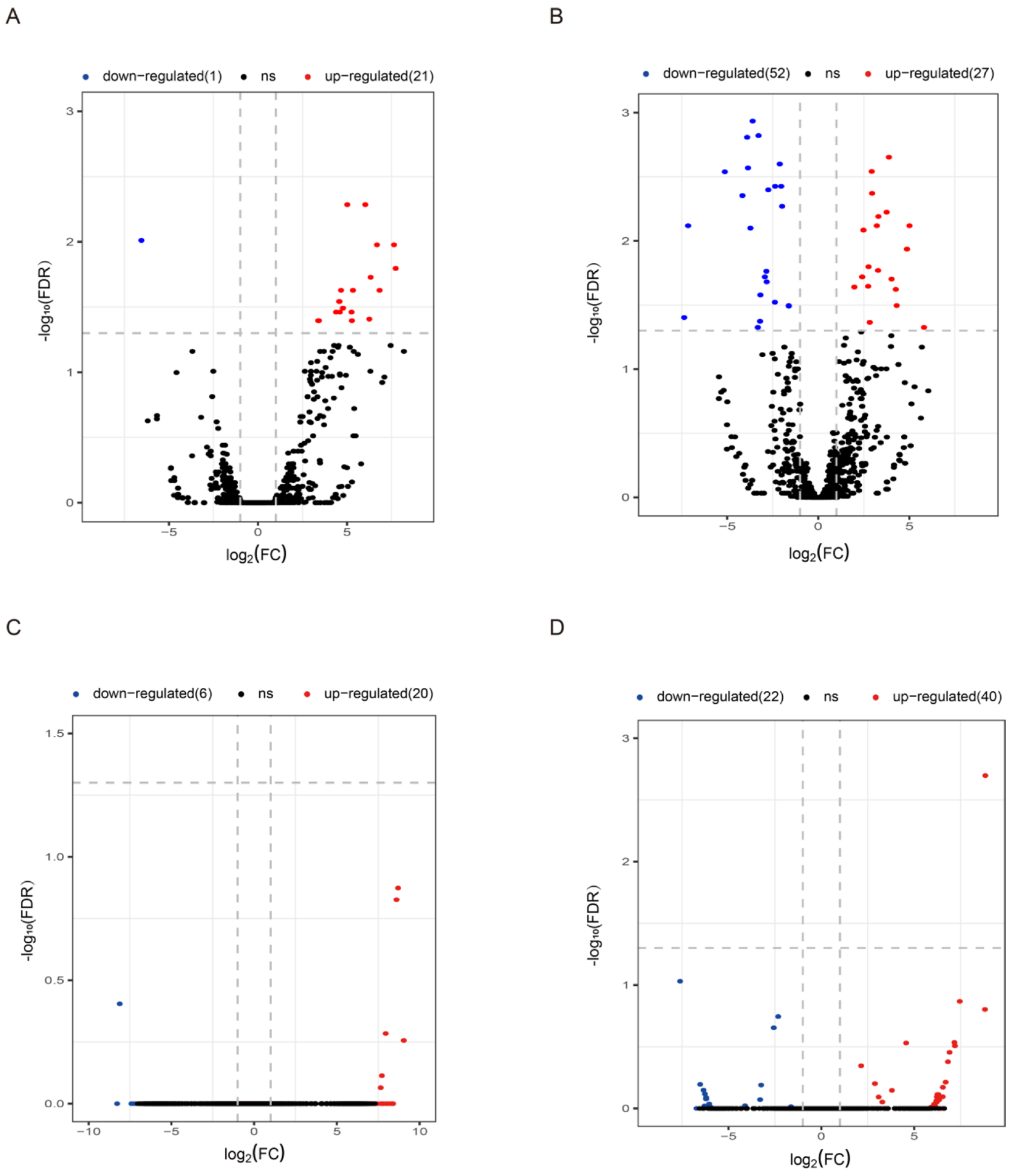

Supplementary Figure 3: Differentially expressed miRNA and circRNA during aging. (**A**) Differentially expressed miRNA in OYCM (old cardiac muscle VS young cardiac muscle), the red dots and blue dots respectively represent up-regulated and down-regulated mRNAs during aging. (**B**) Differentially expressed miRNA in OYSM (old skeletal muscle VS young skeletal muscle). (**C**) Differentially expressed circRNA in OYCM. (**D**) Differentially expressed circRNA in OYSM.

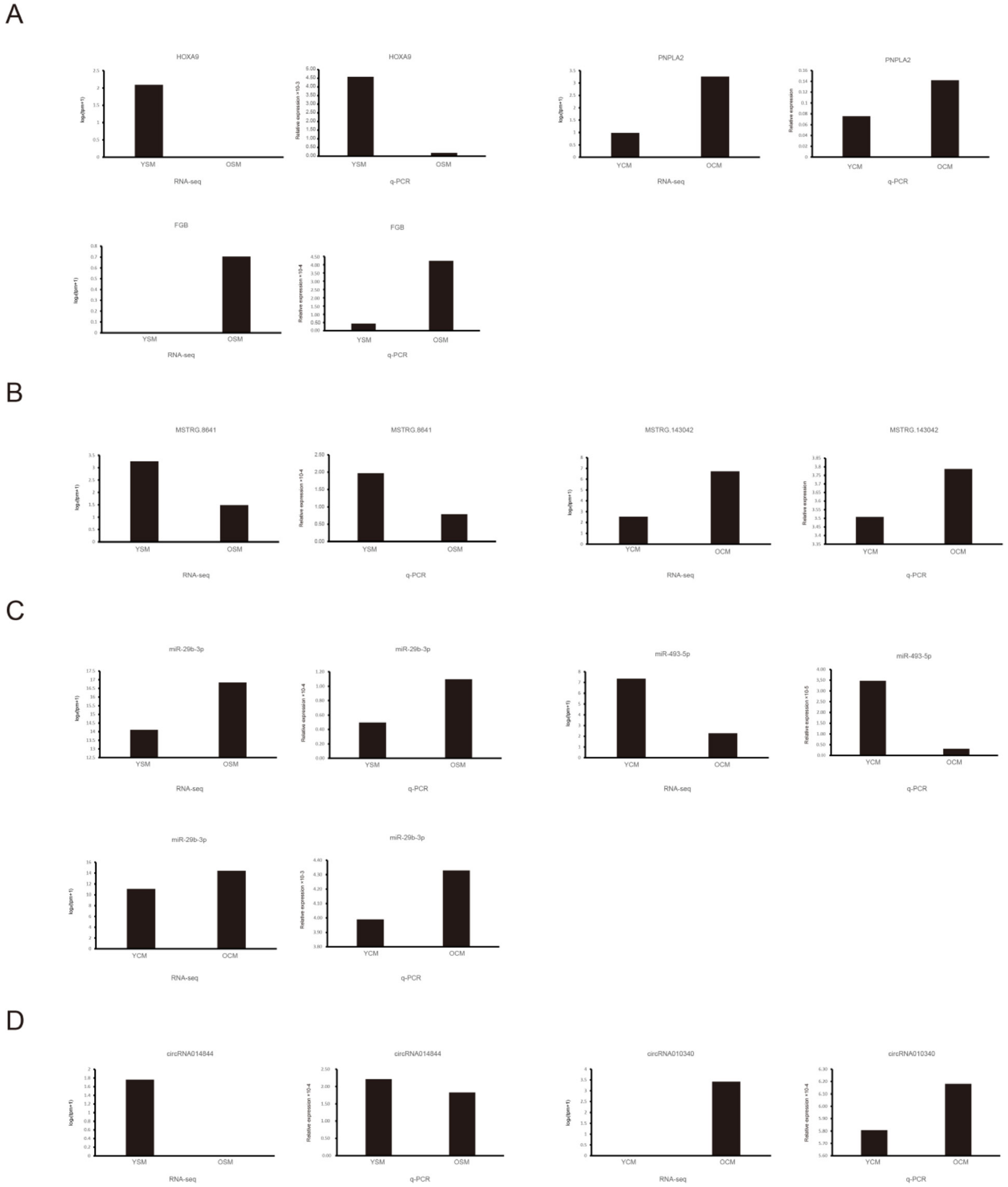

Supplementary Figure 4: Validation of differentially expressed genes. (**A**) Validation of selected differentially expressed mRNAs. (**B**) Validation of selected differentially expressed lncRNAs. (**C**) Validation of selected differentially expressed miRNAs. (**D**) Validation of selected differentially expressed circRNAs. "YCM" is short for "young cardiac muscle", "OCM" is short for "old cardiac muscle"; "YSM" is short for "young skeletal muscle", "OSM" is short for "old skeletal muscle".

Supplementary Table 1: Obtained clean data for RNA sequencing

Sample ID	Reads number (M)	Data size (G)	Mapping rate (%)	GC (%)	Q30 (%)
YCM-1	62.86	9.43	65.5%	45	92.79
YCM-2	68.24	10.24	69.7%	46	92.79
OCM-1	75.58	11.34	67.2%	43	93.48
OCM-2	69.19	10.38	71.8%	49	95.68
YSM-1	83.96	12.59	68.5%	53	95.3
YSM-2	74.15	11.12	69.5%	53	95.31
OSM-1	80.19	12.03	70.6%	51	93.46
OSM-2	62.98	9.45	67.7%	54	92.71

Supplementary Table 2: Obtained clean data for small RNA sequencing

Sample ID	Reads number	Mapped reads	Mapping rate (%)	Q30 (%)
YCM-1	10,953,027	8,149,216	74.40	97.92
YCM-2	9,734,686	7,554,101	77.60	97.86
OCM-1	11,407,462	8,295,659	72.72	97.00
OCM-2	11,035,054	9,485,281	85.96	97.82
YSM-1	9,248,915	8,349,084	90.27	98.26
YSM-2	15,202,241	13,293,605	87.45	98.19
OSM-1	12,138,912	10,228,412	84.26	97.18
OSM-2	17,005,570	15,121,275	88.92	97.30

Supplementary Table 3: Primer sequences for qPCR

Genes	Forward	Reverse	Tm (°C)
HOXA9	5′-GCATTAAACCTGAACCGCTGTC-3′	5′- AACTGGAGGAGAACCACAAGCA-3′	63
PNPLA2	5′- ATGTCATCATAACCCGCTTCG-3′	5′- GTTGTCTGAGATGCCACCGTC-3′	63
FGB	5′- GATCTCGCTGAGCAAATACAAGG-3′	5′- TCGTAGGTGCTGAAGAACATGCT-3′	63
GAPDH	5′- TTCCAGTATGATTCCACCCACG-3′	5′- CCATTTGATGTTGGCGGGAT-3′	63
ACTB	5′- CTGCGGCATCCACGAAACT-3′	5′- GTGATCTCCTTCTGCATCCTGTC-3′	63
MSTTRG.8641	5′- GCGCAGACAGAGCTATTCCAG-3′	5′- AGGGTGTTCAAAGTAGTCAAGGGA-3′	63
MSTTRG.143042	5′- TGTCGGTTCTTCCCGCACT-3′	5′- CGTCCATCTTTCCACCCTCAT -3′	63
miR-29b-3p	5′- GGTAGCACCATTTGAAATCAGTGTT -3′	Universal primer supplied by kit	63
miR-493-5p	5′- GGCTTGTACATGGTAGGCTTTCAT -3′	Universal primer supplied by kit ‘	63
U6	5′-GGAACGATACAGAGAAGATTAGC-3′	5′-TGGAACGCTTCACGAATTTGCG-3′	63
circRNA014844	5′-AAACAAGCCATGGAGAGGTTACTAG-3′	5′-TCCGAGTGTCCTTCCACAGAGT-3′	60
circRNA010340	5‘-GTCTCTTTGCAGCAACACTCAG-3′	5′-AAGAAACGGAACAGTCACTCAAGA-3‘	60

Suppelemntary Dataset 1: Identified genes in our data. See_ Suppelemntary_Dataset 1

Suppelemntary Dataset 2: Identified tissue-specific expressed genes in our data. See_ Suppelemntary_ Dataset 2

Suppelemntary Dataset 3: Clustering of special expressed genes in cardiac muscle and skeletal muscle. See_ Suppelemntary_Dataset 3

Suppelemntary Dataset 4: Differentially expressed genes during cardiac muscle and skeletal muscle aging. See_ Suppelemntary_Dataset 4

Suppelemntary Dataset 5: Gene Ontology analysis for clustering of special expressed genes. See_ Suppelemntary_Dataset 5

Suppelemntary Dataset 6: Gene Ontology analysis for differential expression genes. See_ Suppelemntary_Dataset 6

Suppelemntary Dataset 7: Gene Ontology analysis for differentially expressed lncRNAs. See_ Suppelemntary_Dataset 7

Suppelemntary Dataset 8: Co-expression of differentially expressed miRNAs and differentially expressed circRNAs. See_ Suppelemntary_Dataset 8
